# Dialdehyde Starch Nanocrystals as a Novel Cross-Linker for Biomaterials Able to Interact with Human Serum Proteins

**DOI:** 10.3390/ijms23147652

**Published:** 2022-07-11

**Authors:** Katarzyna Wegrzynowska-Drzymalska, Kinga Mylkie, Pawel Nowak, Dariusz T. Mlynarczyk, Dorota Chelminiak-Dudkiewicz, Halina Kaczmarek, Tomasz Goslinski, Marta Ziegler-Borowska

**Affiliations:** 1 Department of Biomedical Chemistry and Polymer Science, Faculty of Chemistry, Nicolaus Copernicus University in Torun, Gagarina 7, 87-100 Torun, Poland; kasiawd@doktorant.umk.pl (K.W.-D.); kinga.mylkie@doktorant.umk.pl (K.M.); nowak19981411@wp.pl (P.N.); dorotachd@umk.pl (D.C.-D.); halina@umk.pl (H.K.); 2 Chair and Department of Chemical Technology of Drugs, Poznan University of Medical Sciences, Grunwaldzka 6, 60-780 Poznan, Poland; mlynarczykd@ump.edu.pl (D.T.M.); tomasz.goslinski@ump.edu.pl (T.G.)

**Keywords:** dialdehyde starch nanocrystals, cross-linking, Microtox test, HSA and AGP adsorption

## Abstract

In recent years, new cross-linkers from renewable resources have been sought to replace toxic synthetic compounds of this type. One of the most popular synthetic cross-linking agents used for biomedical applications is glutaraldehyde. However, the unreacted cross-linker can be released from the materials and cause cytotoxic effects. In the present work, dialdehyde starch nanocrystals (NDASs) were obtained from this polysaccharide nanocrystal form as an alternative to commonly used cross-linking agents. Then, 5–15% NDASs were used for chemical cross-linking of native chitosan (CS), gelatin (Gel), and a mixture of these two biopolymers (CS-Gel) via Schiff base reaction. The obtained materials, forming thin films, were characterized by ATR-FTIR, SEM, and XRD analysis. Thermal and mechanical properties were determined by TGA analysis and tensile testing. Moreover, all cross-linked biopolymers were also characterized by hydrophilic character, swelling ability, and protein absorption. The toxicity of obtained materials was tested using the Microtox test. Dialdehyde starch nanocrystals appear as a beneficial plant-derived cross-linking agent that allows obtaining cross-linked biopolymer materials with properties desirable for biomedical applications.

## 1. Introduction

Natural polymers are increasingly being used in medical sciences, pharmaceutical, and food industries due to their high biocompatibility and biodegradability. They are also susceptible to numerous functionalizations due to multiple reactive groups, such as hydroxyl and amino groups. Chitosan and gelatin are examples of such materials. Chitosan is non-toxic, bioactive, and antibacterial [1]. This significantly increases the interest in research where chitosan is used in tissue engineering [2], drug delivery systems [3], wound dressing materials [4], packaging materials [5], and as a dietary supplement in weight reduction and preparations lowering cholesterol [6]. Another biomaterial commonly used in biomedical applications is gelatin. Gelatin is one of the main biopolymers widely used in the food, cosmetic, biomedical, and pharmaceutical industries [7]. However, pure gelatin generally has uncontrollable dissolvability and degradability, poor mechanical properties, and thermal instability, which significantly limit its wide practical applications [8]. Polysaccharides require cross-linking to improve their mechanical strength and stability in an aqueous environment. It can be done by physical and chemical methods, e.g., UV irradiation [9], the use of natural and synthetic cross-linkers [10,11], or enzymes [12]. Compared to the other methods, the main advantage of chemical cross-linking is the formation of a strong covalent bond [13]. The standard cross-linker used for biomedical applications is glutaraldehyde. However, this compound cannot be widely used due to inherent disadvantages, especially in biomedical applications. It was found that glutaraldehyde can lead to undesirable effects of cytotoxicity.

Moreover, glutaraldehyde is corrosive and irritating to the skin, eyes, and respiratory tract and is considered to cause health problems for those who handle it, e.g., occupational asthma [14,15]. Lee et al. [16] showed a decrease in mitochondrial activity in nanofiber scaffolds cross-linked with glutaraldehyde after two days of culture. Rho et al. [17] also observed similar cytotoxicity behavior when using glutaraldehyde to cross-link collagen nanofibers. Current trends aim to look for non-toxic and safe cross-linkers, e.g., based on biopolymers, which can meet high requirements in medicine [18].

In the past few decades, intensive research on the improvement of the properties of polysaccharides resulted in the preparation of nanocrystalline polysaccharides. They are characterized by many valuable properties, such as high tensile strength, differentiated morphology, and large specific surface area, distinguishing them from inorganic nanoparticles [19]. In starch granules, the crystalline and amorphous regions coexist. Starch nanocrystals (NCSs) are crystalline structures resulting from the disruption of the amorphous form of starch granules by acid hydrolysis [20]. Recently, nanostarch has gained much attention as a potential reinforcing material in composites due to its excellent mechanical, biodegradable, renewable, and biocompatible properties. Starch nanocrystals, which combine natural abundance and excellent mechanical properties, are promising candidates as polymer reinforcement agents, e.g., natural rubber [21] and soy protein plastics [22].

**Figure 1:**
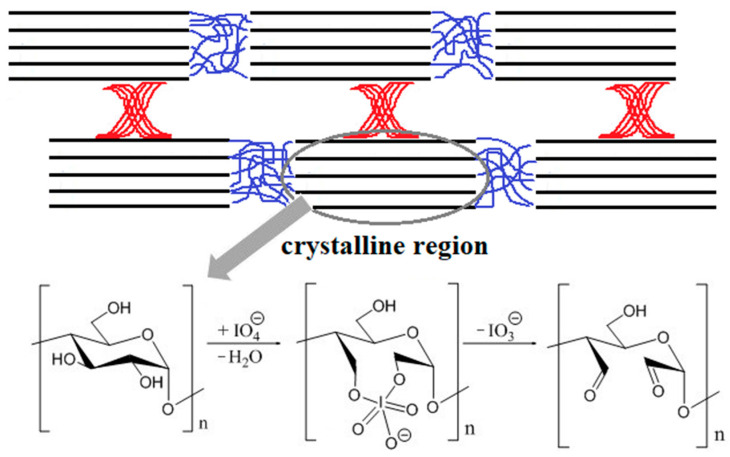
Scheme of the oxidation of nanocrystalline starch with sodium periodate.

Nowadays, numerous studies are focused on the topic of modification of nanocrystalline polysaccharides. The periodate oxidation process is one of the methods of modification of nanocrystalline polysaccharides. In this process, the bond between C2 and C3 in the glycosidic ring is cleaved, and then two aldehyde groups are introduced to these carbon atoms [23]. This simple one-pot method requires only sodium periodate and water as the oxidant and solvent, respectively (Figure 1). The aldehyde groups introduced into the polysaccharide structure can serve as cross-linking agents for biopolymers. The dialdehyde polysaccharides based on pectin [24], chitosan [25], xylan [26], alginate [27], starch [28], hyaluronic acid [29], pullulan [30], galactomannan [31], cellulose [32], carboxymethyl cellulose [33], and xanthan gum [34] are used for cross-linking of polymers containing amino groups.

Proteins play a crucial role in the functioning of living cells. Research into the adsorption of proteins on the surface of materials has gained widespread attention in various applications [35]. Therefore, it is necessary to study the interaction of multiple types of proteins on the surface of biofilms. Examples of such proteins are human serum albumin (HSA) and acid glycoprotein (AGP). HSA is the most abundant globular protein in blood plasma, with a physiological concentration of 40–50 g/L. This blood serum protein interacts with many drugs, making it widely used in the clinical treatment of diseases [36]. Acid glycoprotein is a member of the acute phase protein family with a total molecular weight of 41–44 kDa [37]. It has a single polypeptide chain of 183 amino acid residues and is synthesized by the liver and organs such as the heart and lungs [38]. The primary physiological function of AGP is to transport compounds such as basic drugs, heparin, and certain steroid hormones [39].

To the best of our knowledge, there is only published work concerning the application of dialdehyde cellulose nanocrystals as cross-linking agents [40,41], but not about dialdehyde starch nanocrystals. Chen et al. [42] described the synthesis method of oxidized starch nanocrystals. They reported that the oxidation reaction time was two hours in the dark, and the suspensions were washed by centrifugation with distilled water to pH = 7. A subsequent paper by Chen et al. [43] proposed combining nanocrystalline dialdehyde starch with graphene oxide nanosheets to form highly porous aerogels, which can be used as supercapacitor electrodes and efficient adsorbents. However, no research group used nanocrystalline dialdehyde starch as a cross-linking agent. Oxidized starch nanocrystals, like starch nanocrystals, have a large specific surface area [44] of the material, which could affect the efficiency of the cross-linking process. In addition, starch nanocrystals are characterized by good mechanical properties. Hence, they are used as reinforcement agents [45]. Therefore, we consider that dialdehyde starch nanocrystals can also improve the mechanical properties of materials. In addition, the nanocrystalline form of oxidized starch might be a more favorable cross-linking agent for the properties of the obtained material.

The objective of the present study was to prepare dialdehyde starch nanocrystals for cross-linking of biopolymer films. Commonly available methods were applied to perform the detailed characterization of all received materials. In addition, the mechanical properties of the obtained materials and their hydrophilic nature using contact angle measurement were determined. The acute toxicity of the prepared biofilms was preliminarily studied using the Microtox test. The biological properties of designed, cross-linked biopolymer films, including adsorption of protein (human serum albumin (HSA) and acid glycoprotein (AGP)), were tested.

## 2. Results and Discussion

### 2.1. Synthesis of Starch Nanocrystals

The acid hydrolysis method of native corn starch was used to synthesize starch nanocrystals. This simple method removes the amorphous region of polysaccharides using sulfuric acid. The differences in the size of the granules, their porosity, and the amylose content affect the hydrolysis efficiency. In addition, it was reported that the pores on the starch surface increase the availability of acid to the inside of the granule, which promotes the hydrolysis process [46]. The reaction yield of the obtained nanocrystalline starch was 26%. The NCS yield from maize starch was the same as that reported for waxy maize [47] and similar to that reported for mango kernel starch [48].

### 2.2. Characterization of Starch Nanocrystals

**Figure 2:**
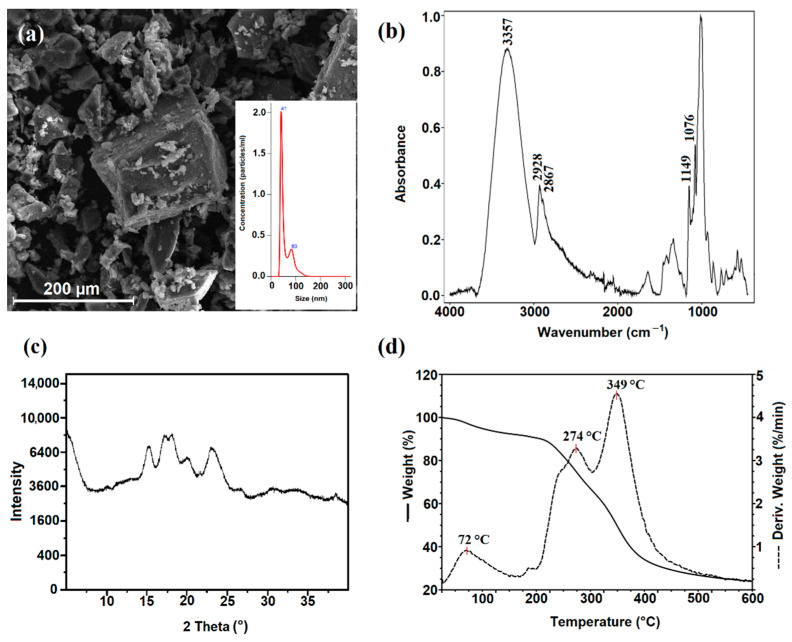
(**a**) SEM image, with inset showing the size distributions and particle concentrations of starch nanocrystals; (**b**) ATR-FTIR spectra; (**c**) X-ray diffraction (XRD) patterns; and (**d**) TGA-DTG curves of starch nanocrystals.

The size distributions and particle concentrations of NCSs are shown in Figure 2a. The mean size of the starch nanocrystals was 58 nm and was consistent with previous results described by other research groups [49,50].

The structure of NCSs was confirmed by ATR-FTIR analysis (Figure 2b). Starch nanocrystals displayed a characteristic FTIR spectrum like that of the native starch. In the spectrum of NCSs, the absorption bands at 3357 cm^−1^ attributed to O–H stretching vibrations, 2867 cm^−1^ to asymmetric CH stretching, 1149 cm^−1^ to the stretching vibration absorption peak of C–O, and 1076 cm^−1^ to C–O stretching were observed. The received spectrum is in agreement with the literature data [51]. The band’s intensity at 2928 cm^−1^, corresponding to the C-H stretching vibration, was found to change with the ratio of amylose to amylopectin [52]. A reduction in the amylose ratio results increased the intensity of this band (2928 cm^−1^) in the ATR-FTIR spectrum. In the spectrum obtained for starch nanocrystals, an increase in the intensity of this characteristic band was observed, which confirms selective hydrolysis of the amorphous regions, mainly containing amylose.

The X-ray diffraction signal of NCSs (Figure 2c) was observed at 2θ around 15°, a doublet one at 17–18°, and another near 23°, corresponding to a typical A-type starch pattern [53]. Similar observations were previously reported for starch nanocrystals derived from cassava starch [54].

The SEM images of NCSs are shown in Figure 2a. After acid treatment, the corn starch granules were fragmented to nanocrystals, with small, irregular, and square-like structures with larger aggregates. The morphology of starch nanocrystals is in good agreement with the crystalline type, as previously reported [55]. Nanocrystals produced from A-type starches rendered square-like particles.

A TGA-DTG analysis was performed to determine the thermal properties of starch nanocrystals. The TGA curve and its derivative for starch nanocrystals are shown in Figure 2d. The thermogravimetric curve of NCSs shows three degradation stages. In the range between 30 °C and 140 °C, a 7% mass loss was observed due to evaporation of the adsorbed water. The intensive decomposition takes place between 310 °C and 450 °C, with a 41% mass loss, corresponding to the thermal degradation of the starch polymer chain. The additional decomposition step of nanocrystalline starch in the temperature range of 120–360 °C, with 27% weight loss, due to the partial degradation of the NCS structure was observed.

### 2.3. Synthesis of Dialdehyde Starch Nanocrystals

The oxidized starch nanocrystals were synthesized by the oxidation reaction of the previously obtained starch nanocrystals. The common oxidation reaction of starch to dialdehyde starch is described. Still, only two reports are available in the literature on the synthesis and potential utility of oxidized starch nanocrystals. Sodium periodate (NaIO_4_) was used as an oxidation agent in a 1:1 weight ratio with starch nanocrystals to give dialdehyde starch nanocrystals (NDASs) as high as 65% degree of oxidation. The reaction was carried out at 40 °C for 3 h. Oxidized starch nanocrystals were precipitated from the reaction mixture with acetone, which greatly facilitated its separation. The reactions with different sodium periodate:nanocrystal starch ratios (0.5:1, 0.7:1) were also tested, resulting in 30% and 45% oxidation degrees.

### 2.4. Characterization of Dialdehyde Starch Nanocrystals

**Figure 3:**
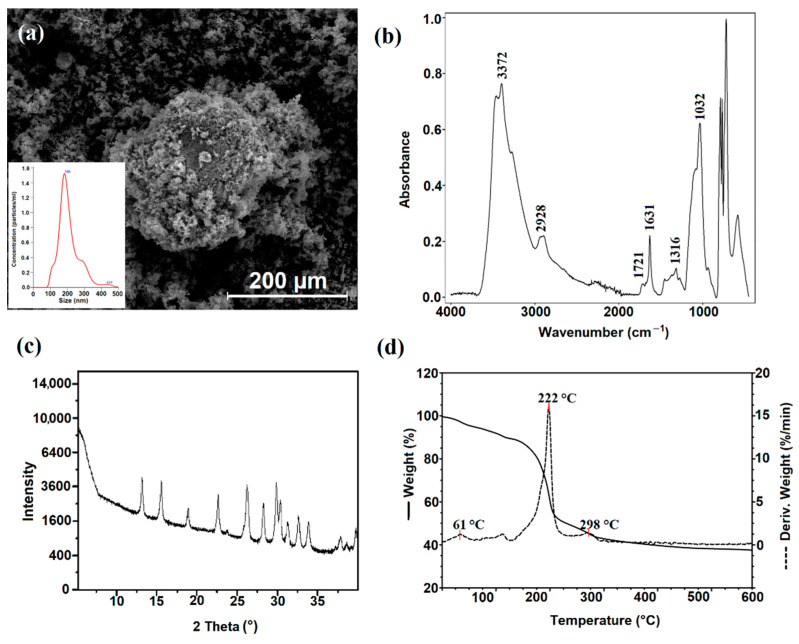
(**a**) SEM image, with inset showing the size distributions and particle concentrations of dialdehyde starch nanocrystals; (**b**) ATR-FTIR spectra; (**c**) the X-ray diffraction (XRD) patterns; and (**d**) the TGA-DTG curves of dialdehyde starch nanocrystals.

The size distributions and particle concentrations of NDASs are shown in Figure 3a. The mean size of the oxidized starch nanocrystals was 212 nm. The oxidation process increased the size of the dialdehyde starch nanocrystals. Opposite results of particle size changes were reported by Chen et al. [42]. 

The structure of NDASs was confirmed by ATR analysis. After oxidation, the peak appearing at 1721 cm^−1^ was attributed to the characteristic absorption of carbonyl groups (Figure 3b). Its intensity was very weak, possibly because internal cross-links created some hemiacetal linkage during the oxidation process. The new sharp band at 1631 cm^−1^ was observed. It may be derived from the carbonyl group after an effective oxidation process of starch nanocrystals. Additionally, the band at 3372 cm^−1^ representing hydroxyl stretching vibrations was of lower intensity than the NCS spectra. This can be explained by the opening of cyclic structure and oxidation of starch saccharide units. Significant changes were also noticed in the vibration range of 1300–1400 cm^−1^, which may be caused by the opening and oxidation in the glucoside ring between the second and third carbon atoms.

The X-ray diffraction patterns of NDASs showed many intensive signals (2θ = 13.2°, 15.6°, 22.7°, 26.3°, 30.0°, 31.4°, 32.8°, and 34.1°) proving the presence of the crystalline nature of the obtained product. After careful analysis of the literature data, it was shown that the above diffraction signals could be attributed to the signals derived from NaIO_3_. It should be pointed out that obtained product was repeatedly washed with deionized water, and despite this, NaIO_3_ signals in the diffractogram of NDASs were observed. This may indicate the formation of a strong complex between the dialdehyde product and the reduced form of the oxidant. Previous work noted similar mechanisms for dialdehyde chitosan and dialdehyde starch [56,57].

After oxidation, the regular square-like particles of starch nanocrystals disappeared and were transformed into rough shapes with many needle forms (Figure 3a). The needle form length was less than 10 µm, and coarse shapes had an average size of 5–20 µm.

To determine the thermal properties of oxidized starch nanocrystals, a TGA-DTG analysis was performed under the same conditions as in the case of NCSs. The TG curve of NDASs shows three degradation stages (Figure 3d). The first degradation step is observed between 20 °C and 80 °C and shows a 5% loss of weight. This weight loss may be related to the loss of bound water. The intensive destruction takes place in the second stage in the range between 156 and 410 °C and is associated with the 49% mass loss corresponding to the thermal degradation of the dialdehyde starch nanocrystal chain. At the maximum decomposition rate, T_max_ = 222 °C in oxidized starch nanocrystals is lower than that for starch nanocrystals (T_max_ = 365 °C) due to the fact that nanocrystalline oxidized starch has open pyranose rings after oxidation reaction on two and three of the carbon atoms in this structure. The same effect was observed with dialdehyde starch.

### 2.5. Formation of Chitosan, Gelatin, and Chitosan–Gelatin Films by Cross-Linking with Dialdehyde Starch Nanocrystals

The primary purpose of this research work was to obtain innovative polysaccharide-based material biofilms for biomedical applications. To investigate the effect of oxidized starch nanocrystals as a cross-linking agent, biofilms of chitosan, gelatin, and chitosan–gelatin (1:1) were prepared. The amount of cross-linker was 5 wt%, 10 wt%, and 15 wt% in relation to chitosan, gelatin, and chitosan–gelatin. Biopolymer films were smooth and transparent (Figure S1).

In order to determine the influence of oxidized starch nanocrystals on the properties of biofilms, the structure, morphology, thermal stability, and swelling ability were characterized for obtained materials. The properties of the obtained materials determine their subsequent applications. Materials for biomedical applications should be non-toxic and biocompatible because of their interaction with biological membranes. They should also be characterized by hydrophilicity, adequate strength, and porosity, which will allow the free growth of cells on the material surface.

#### 2.5.1. Determination of Cross-Linking Degree of the Films

**Figure 4:**
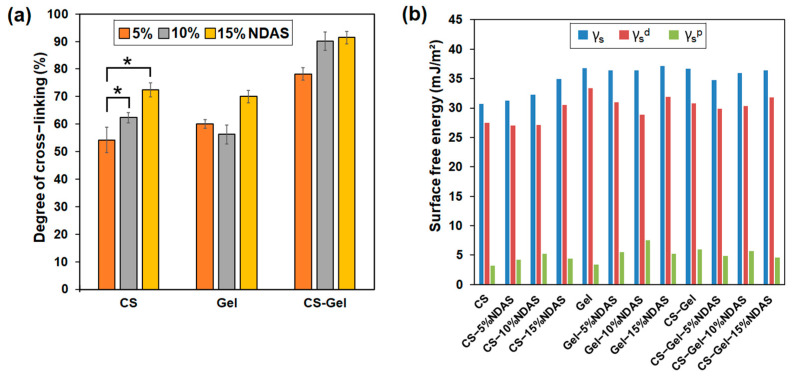
(**a**) Degree of cross-linking of chitosan (CS), gelatin (Gel), and chitosan–gelatin (CS-Gel, 1:1) cross-linked by 5%, 10%, and 15% cross-linker; statistical significance is indicated with asterisks: * *p* < 0.05. (**b**) The surface free energy (γ_s_) and its polar (γ_s_^p^), and dispersive (γ_s_^d^) components for biofilms based on chitosan (CS), gelatin (Gel), and chitosan–gelatin (CS-Gel) cross-linked with 5%, 10%, and 15% NDASs.

The cross-linking of macromolecules causes significant changes in the polymeric material properties. Thus, the degree of cross-linking is an essential and important feature for polymer networks. Cross-linking improves the thermal stability and resistance to cracking effects by liquids. In determining the degree of cross-linking, the non-cross-linked film was dissolved completely in an acetic acid solution (5%), and the degree of cross-linking of studied films is presented in Figure 4a. As expected, the degree of cross-linking of the films increases with the increase in the amount of cross-linking agent. The chemical cross-linking between components of films and dialdehyde starch nanocrystals improved the cross-linking density of polymer films. The highest degree of cross-linking was achieved for chitosan–gelatin films cross-linked with 15% NDAS addition, which may be caused by intermolecular cross-linking between both biopolymers.

#### 2.5.2. Contact Angle Measurement

Interactions between materials and biological fluids are defined by surface free energy. Measurements of the contact angle of polar (glycerin) and non-polar (diiodomethane) liquids can assess the resulting materials’ surface nature and potential applications. Surface free energy is one of the parameters determining the applications of the obtained materials. The dispersive and polar components provide detailed information on the tested surface. The contact angle of glycerin and diiodomethane and the surface free energy and its polar and dispersive components are presented in Table S1 and Figure 4b.

Results prove that all obtained biofilms have average glycerin contact angles lower than 90°, indicating a hydrophilic surface. The value of the surface free energy of pristine chitosan is 30.70 mJ/m^2^, which is well confirmed by literature data [58]. Moreover, the chitosan films cross-linked with NDASs were characterized by a lower wettability by glycerol than pure chitosan. Chitosan and gelatin films cross-linked by NDASs were characterized by slightly higher surface free energy values than pure chitosan and pure gelatin. The same situation may be observed for the values of the polar component for neat chitosan and gelatin films after the cross-linking process. Gelatin and chitosan films cross-linked by NDASs were characterized by higher values of γ_s_^p^ than the neat gelatin and chitosan film. The highest polarity among the selected materials was Gel-10%NDAS due to it having the highest polar component of surface free energy. For chitosan–gelatin materials cross-linked with NDASs, a decrease in surface free energy and its polar component was observed.

Gierszewska et al. [59] investigated the effect of adding glutaraldehyde (0.5 wt% content) to chitosan and chitosan/montmorillonite to determine changes in hydrophilicity. They noticed that the application of the cross-linking agent caused a decrease in the surface free energy, from 33.5 mJ/m^2^ (pure chitosan) to 29.5 mJ/m^2^ in chitosan with glutaraldehyde. Similar results were obtained for chitosan/montmorillonite cross-linked with dialdehyde starch. The addition of a cross-linker to these samples caused a decrease in surface free energy. In work on chitosan and mixtures of collagen materials, hyaluronic acid, and chitosan cross-linked with oxidized starch, the surface free energy values after the modifications were higher [60].

#### 2.5.3. ATR-FTIR Spectroscopy and X-ray Diffraction (XRD)

**Figure 5:**
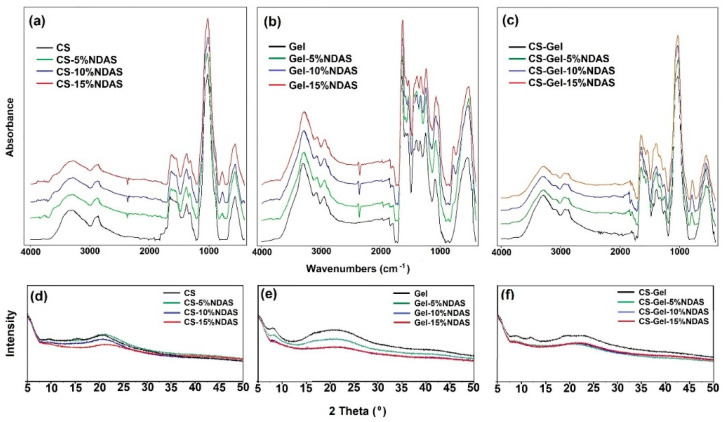
ATR-FTIR spectra of (**a**) chitosan, (**b**) gelatin, and (**c**) chitosan–gelatin films cross-linked by dialdehyde starch nanocrystals and the X-ray diffraction (XRD) patterns of (**d**) chitosan, (**e**) gelatin, and (**f**) chitosan–gelatin films cross-linked with dialdehyde starch nanocrystals.

The strong and broad bands at 3356 cm^−1^ and 3287 cm^−1^ were attributed to stretching vibrations of O–H and N–H, respectively. A weak band at 2867 cm^−1^ is assigned to stretching vibrations of C-H. The characteristic absorption bands at 1649 cm^−1^ (C=O stretching of amide I) and 1551 cm^−1^ (N–H bending of amide II) were observed in the unmodified chitosan spectrum (Figure 5a). The bands in the region of 1151 cm^−1^ to 1027 cm^−1^ were the characteristic bands of C–O–C asymmetrical stretching and confirmed by literature data [61].

The changes in the intensity and shape of the hydroxyl band (3356 cm^−1^) and amine band (3287 cm^−1^) can be explained by the reaction of an amino group of chitosan with the carbonyl group from a cross-linker. Hoffmann et al. obtained similar results for chitosan films cross-linked with dialdehyde dextran and glutaraldehyde [62]. Furthermore, in chitosan films cross-linked with NDASs, a new band near 778 cm^−1^ is observed, attributed to the =C–H bond (deformation stretching vibrations). It’s due to the fact that some aldehyde groups of cross-linker are not linked to chitosan.

In the spectrum of gelatin, the characteristic absorption bands at 3292 cm^−1^ (assigned to the O–H stretching, N–H stretching vibrations, and intramolecular hydrogen bonds), 3076 cm^−1^ (amide B), 2943 cm^−1^ (CH stretching), 1636 cm^−1^ (C=O stretching vibration of amide I), 1537 cm^−1^ (amide II, attributed to a combination of CN stretch and in-plane NH deformation), 1242 cm^−1^ (amide III, corresponds to CN stretching and NH in-plane bending), and 1077 cm^−1^ (C–O stretching) were observed (Figure 5b). The received spectrum is consistent with the literature data [63].

The amino group from gelatin and the aldehyde group of NDASs form a Schiff base, and a new peak can appear in the region 1650–1600 cm^−1^ [64]. In addition, in this bio-film, this cross-linking fingerprint region (1650–1600 cm^−1^) is covered by the strong amide I peak of gelatin [65]. In addition, the band at 1077 cm^−1^ (C–O stretching vibration) becomes very broad with the addition of NDASs.

The ATR-FTIR spectrum of the CS-Gel composite showed similar characteristic peaks of the pure chitosan and gelatin with some shifts. The peaks related to bending vibrations of the amine groups in pure chitosan were shifted from 1649 cm^−1^ and 1551 cm^−1^ to 1642 cm^−1^ and 1573 cm^−1^, respectively, in the CS-Gel composite. The shift of amino bands in the CS-Gel composite spectrum showed a complex involving intermolecular hydrogen bonding between chitosan and gelatin [66].

A new band formation at 779 cm^−1^ for CS-Gel cross-linked with NDASs was also observed, as for cross-linked chitosan samples. In addition, no significant changes in the spectrum of the cross-linked CS-Gel film were noticed.

The crystalline structure of CS, Gel, and the CS-Gel films cross-linked by NDASs was characterized by XRD patterns and is shown in Figure 5d–f. The X-ray diffractograms of CS show crystalline diffraction peaks at 2θ = 10.5°, 15.1°, and 20.9°. According to the literature [67], these crystalline peaks of pure CS can be ascribed to the reflection planes of (020), (110), and (200). All the chitosan films cross-linked with NDASs showed diffraction peaks at 2θ = 20.9°, while the intensity of this peak decreased with the increase in the cross-linker amount. The peaks at 2θ = 10.5° and 15.1° for CS-15%NDAS disappeared. For gelatin, the signal located at about 2θ  =  8.6° corresponds to the inter-helix distance of the triple helix of gelatin, and a broad diffraction peak in the 2θ range of 15.0–25.0 is typical of the amorphous fraction of gelatin [68]. The obtained pattern is consistent with the literature data [69]. For the gelatin samples, an increase in the amount of cross-linking agents caused a decrease in the intensity of signals. However, the chitosan/gelatin mixture did not show any crystalline peaks, indicating the amorphous structures of chitosan and gelatin. This result also exhibited that the chitosan and gelatin were mixed well, forming the composite [70]. The intensity of the diffraction patterns for CS-Gel cross-linked by NDASs decreases with an increasing amount of cross-linking agent. The same effect was observed for cross-linked gelatin and chitosan films.

#### 2.5.4. Thermal Analysis

The thermogravimetric decomposition process was used to define the thermal properties of the obtained biofilms. The thermal parameters of the obtained biofilms are listed in Table 1, and the TG and DTG curves are presented in Figure S2.

The thermal stability of the films was analyzed using thermogravimetric analysis (TGA) and the derived thermogravimetric analysis (DTG) to observe various stages of degradation. Chitosan film exhibits two degradation stages well explained in the literature [71]. At the first stage of chitosan degradation, approximately 8% of the initial weight loss occurred between 29 and 140 °C due to the loss of moisture content. The main decomposition stage appeared in the temperature range of 180–420 °C. The 51% weight loss in this stage corresponds to main chain scission, side group abstraction, and ring-opening reactions. Gelatin film shows two degradation stages [72]. Neat gelatin shows a main decomposition in the temperature range of 200–450 °C, with 62% weight loss. The 9% initial weight loss between 20 and 120 °C is attributed to the removal of moisture and other volatile impurities. Non-cross-linked chitosan–gelatin film degraded in two steps [73]. The 15% weight loss in the first stage (in the temperature range from 20 to 140 °C) was attributed to water loss. The second stage, with 57% weight loss from 150 to 525 °C, was due to the actual decomposition of the macromolecules.

Three degradation steps were observed in thermogravimetric curves of chitosan, gelatin, and chitosan–gelatin films cross-linked with NDASs. For all cross-linked films, the first decomposition stage ranges between 30 °C and 120 °C. It shows about 8–13% loss in weight due to the evaporation of bound and adsorbed water, similar to unmodified polysaccharides and their mixtures. The main step of thermal degradation of all cross-linked materials is the intensive destruction, which occurs in the temperature range between 160 °C and 350 °C and shows about 55% loss in weight. Furthermore, there is an additional stage of decomposition in the temperature range between 120 °C and 190 °C. The weight loss at this stage is slight (2–4%). It may indicate that the cross-linking bond of biofilms was broken in this step. The content of residue at 600 °C correlates with the range of 23–40%.

Other research groups also investigated the effect of cross-linking agents on the thermal properties of chitosan, gelatin, and their mixtures. Sutirman et al. studied poly (methacrylamide) grafted chitosan beads cross-linked with glutaraldehyde [74], and they also observed three decomposition stages in chitosan cross-linked beads. They observed that the thermal stability of chitosan had been improved through cross-linking and grafting copolymerization. In the work of Kaczmarek-Szczepańska et al. [75], glyoxal (10 wt%) was a cross-linking agent for chitosan hyrogels loaded with tannic acid. After modification, the three steps of degradation of the hydrogel were also noticed. Zhao et al. studied gelatin hydrogels cross-linked with two cross-linking agents (genipin and polyphenol) [76]. They noticed two decomposition stages of samples and observed that the interactions between gelatin and cross-linkers improved the thermal stability. Similar observations have been made by Nieto-Suárez et al. [77] for thermograms of chitosan–gelatin scaffolds cross-linked with glutaraldehyde, and these authors also indicated that chitosan–gelatin materials decompose in three stages. Moreover, they observed that the T_max_ shifts to higher temperatures upon the increase in gelatin concentration, thus indicating higher stability of scaffolds with gelatin.

#### 2.5.5. SEM and AFM

**Figure 6:**
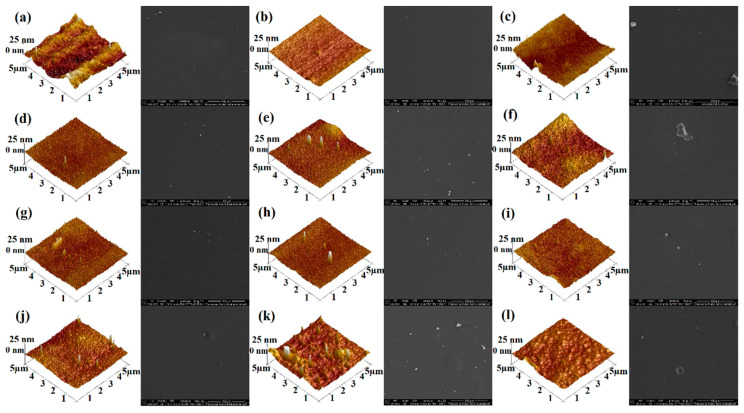
AFM and SEM (1000×) images of (**a**) chitosan films cross-linked by (**d**) 5% NDASs, (**g**) 10% NDASs, and (**j**) 15% NDASs; (**b**) gelatin film cross-linked by (**e**) 5% NDASs, (**h**) 10% NDASs, and (**k**) 15% NDASs; and (**c**) chitosan–gelatin films cross-linked by (**f**) 5% NDASs, (**i**) 10% NDASs, and (**l**) 15% NDASs.

The surface morphology of films was determined by scanning electron microscopy (SEM) for all samples (Figure 6). All the neat films were transparent, smooth-surfaced, and homogeneous. When NDASs were added, the film surfaces became a little rougher in a few samples. However, in most biofilms, the cross-linking process creates a smooth surface, which proves the homogeneity of the samples. The surface structures of the different films depend mainly on the degree of cross-linking and interactions between polymers [78].

The surface roughness is an essential parameter in subsequent material applications. The surface roughness of obtained biofilms was examined using a topographical atomic force microscope. The 3D and 2D atomic force microscopy (AFM) images of the obtained biofilms are shown in Figure 6 and Figure S3, respectively. The pure gelatin film was smooth and homogenous. After increasing NDAS content, as shown in Table 2, rougher surfaces were observed in the originally smooth gelatin matrix.

Furthermore, the surface of the gelatin film displayed a surface roughness, of R_q_ value of 1.83 nm, which increased to 4.70 nm as the NDAS addition reached 15%. A relatively smooth morphology was observed for pure chitosan, which is mainly related to the chitosan’s physical properties, such as high intrinsic chain stiffness [79]. Biofilms based on chitosan modified with oxidized starch nanocrystals are characterized by a relatively rough surface. The opposite trend was observed for chitosan–gelatin films cross-linked with NDASs, and surface roughness decreases with the rising content of cross-linking agents for these materials.

Other research groups also investigated the effect of cross-linking agents on chitosan and its mixtures. Olewnik-Kruszkowska et al. studied chitosan films cross-linked with 1 wt%, 2 wt%, and 3 wt% squaric acid [80] and noted that roughness parameters increased with an increased amount of the cross-linker. They reported the highest surface roughness parameters’ values after cross-linking. In the case of chitosan cross-linked by 3 wt% of squaric acid, Rq and Ra are 3.67 nm and 2.95 nm, respectively. Sionkowska et al. [60] investigated the effect of the addition of dialdehyde starch (5 wt% content) for chitosan and mixtures of collagen, hyaluronic acid, and chitosan on the roughness parameters. They observed that the application of the cross-linking agent promoted an increase in the R_q_ parameter, from 3.2 nm (pure chitosan) to 4.8 nm in the case of chitosan cross-linked with dialdehyde starch. Opposite results were obtained for collagen/hyaluronic acid/collagen cross-linked with oxidized starch. The addition of a cross-linker caused a decrease in the R_q_ parameter from 23.0 nm to 7.2 nm. In the work of Abbasi et al. [81] genipin (0.125 wt%, 0.25 wt%, 0.5 wt%, 0.75 wt%, 1 wt%, and 2 wt%) as a cross-linking agent was used for gelatin films. After modification, the observed roughness was lower for materials with genipin than in the case of pure gelatin film.

#### 2.5.6. Mechanical Properties

**Figure 7:**
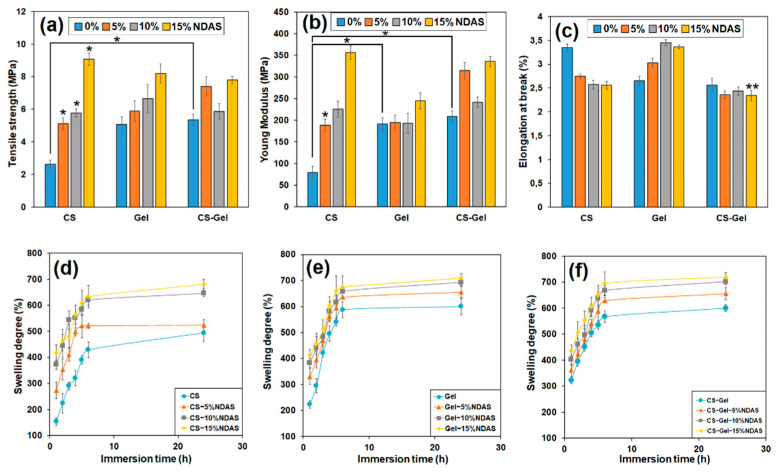
The value of (**a**) tensile strength, (**b**) Young’s modulus, and (**c**) elongation at break of chitosan (CS), gelatin (Gel), and chitosan–gelatin (CS-Gel) cross-linked by 5%, 10%, and 15% cross-linking agent; statistical significance is indicated with asterisks: * *p* < 0.05, ** *p* < 0.01. The swelling degree of (**d**) chitosan, (**e**) gelatin, and (**f**) chitosan–gelatin cross-linked by 5%, 10%, and 15% cross-linker (NDASs).

The mechanical properties are crucial for the applications of biofilms subjected to a certain level of mechanical forces. To investigate the effect of dialdehyde starch nanocrystals on the mechanical properties of biofilms, mechanical tests were performed, and the obtained tensile strength, Young’s modulus, and elongation at break are summarized in Figure 7a–c, respectively.

The values of tensile strength for unmodified samples of chitosan, gelatin, and chitosan–gelatin (1:1) were about 3 MPa, 5 MPa, and 5.5 MPa, respectively (Figure 7a). For chitosan and gelatin films cross-linked by NDASs, the tensile strength increases with the increasing addition of cross-linking agents. The sample CS-15%NDAS is characterized by the most significant difference in tensile strength value. This parameter increased to 9.08 MPa, 3 times higher than the value of this parameter for the pure chitosan sample. Moreover, tensile strength increases with rising amounts of cross-linking agents for chitosan and gelatin films cross-linked with NDASs. An increase in strength for all samples with a 5% addition of cross-linking agent in comparison to neat polysaccharides and mixture was observed. Amino groups from chitosan and gelatin form a covalent bond with carbonyl groups from NDASs. The creation of a new cross-linking network in chitosan films is responsible for the increase in mechanical properties.

Young’s modulus describes the stiffness of obtained materials; for pure gelatin, it was about 192 MPa. The value of this parameter for a neat gelatin sample is almost the same as that for gelatin films cross-linked with 5% and 10% NDASs (Figure 7b). In this series of measurements, the gelatin sample with a 15% addition of NDASs reached the highest value of Young’s modulus—245 MPa. Moreover, for chitosan films cross-linked with NDASs, an increase in the Young’s modulus value is observed with an increase in the amount of cross-linking agent. The chitosan samples cross-linked with a 15% addition of NDASs are characterized by the highest value of Young’s modulus, which is 4.5 times higher than that of neat chitosan film. In the case of a mixture of chitosan and gelatin, cross-linked with NDASs, an increase the value of the Young modulus is observed with increasing amounts of cross-linking agent, except for the sample with 10% NDAS addition.

The elongation at break is the ratio of the length of the specimen after and before the break. The percentage of elongation at break indicates the stretchability of the samples [82]. The highest value of percentage of elongation at break for the neat samples (CS, Gel, and CS-Gel) was achieved for the chitosan sample (3.35%). This parameter for gelatin and chitosan–gelatin is practically the same and was about 2.65% and 2.56%, respectively (Figure 7c). In the case of chitosan materials cross-linked with NDASs, the percentage of elongation decreases with an increasing amount of cross-linking agent. The opposite trend was observed for cross-linked gelatin samples. Furthermore, for the mixture of chitosan and gelatin, the elongation at break is almost the same for all films—neat and cross-linked.

In our previous research, we obtained chitosan film cross-linked with dialdehyde starch (DAS) [56]. The tensile strength value for the chitosan sample cross-linked with 15% DAS was about 6 MPa, whereas the sample with the same amount of NDASs was above 9 MPa. The most significant difference in Young’s modulus value was observed for the CS-15%NDAS sample. This parameter increased to about 350 MPa, above 1.5 times more than that for chitosan film cross-linked with the same amount of DAS. Based on the results, it can be concluded that oxidized starch nanocrystals resulted in a greater improvement in mechanical properties of chitosan compared to the chitosan films cross-linked with DAS.

#### 2.5.7. Swelling Ability

The swelling of biofilms greatly depends not only on the properties of the used polymer but also on the medium of swelling. The polymer structure gives the biofilms the ability to absorb solvent, while the cross-links between the network chains provide the biofilms with the ability to resist uncontrolled biodegradation. The retracting and expanding forces must balance each other to reach swelling equilibrium [83]. The swelling ability of the obtained biofilms in PBS solution was determined and is shown in Figure 7d–f. According to the literature, materials based on biopolymers are easily wettable by polar solvents due to the presence of functional groups capable of interacting with water molecules [84]. This usually results in a high degree of swelling. 

The swelling ratio of the pure chitosan sample after the first hour of immersion in the PBS solution was 153%. Moreover, the swelling ratio for chitosan–gelatin films cross-linked with oxidized starch nanocrystals is 3 times higher than that for chitosan film after the same period. For all films cross-linked by NDASs, the percentage swelling ability increases with increasing immersion time in PBS solution. The highest degree of swelling after 24 h is observed for chitosan–gelatin materials cross-linked with NDASs. This is due to the numerous functional groups in chitosan and gelatin macromolecules capable of absorbing polar solvents. 

Skopinska-Wisniewska et al. [85] obtained similar results with gelatin materials cross-linked by dialdehyde starch. The swelling ratio increased in the initial stage and stabilized after 6 h of immersion in PBS for these samples. Kaczmarek et al. [86] investigated the swelling ability of 3D chitosan–gelatin hydrogels cross-linked with oxidized starch. Their results showed that the higher cross-linker content resulted in higher water absorption than unmodified scaffolds. For the sample 50Gel/50CTS + 5% ST (dialdehyde starch), the swelling ability is about 842%. This results from the sample structure reorganization after the cross-linker addition and the change of pore sizes. After immersion for 1 h for the sample CS/Gel + 5% NDASs, we obtained a swelling ability of about 360%, which may be related to the different structure of the material in the form of a thin film and the nanocrystalline structure of the cross-linking agent.

#### 2.5.8. Toxicity Assessment

**Figure 8:**
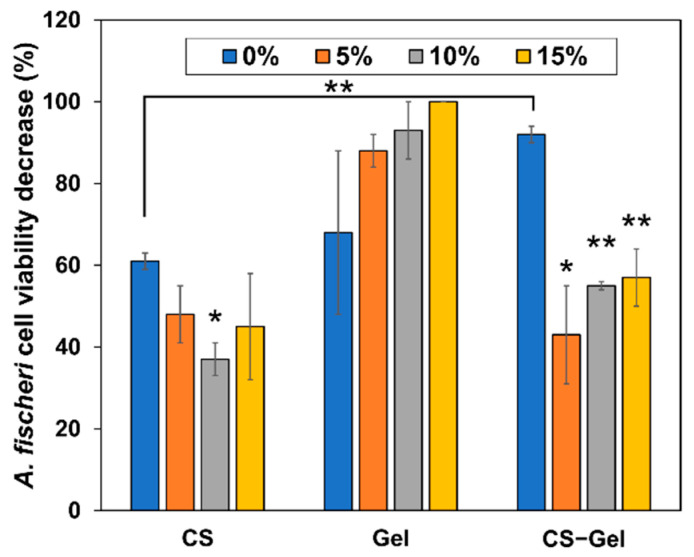
The decrease in *A. fischeri* cell viability upon 5-minute exposure to chitosan (CS), gelatin (Gel), and chitosan–gelatin (CS-Gel) films prepared by cross-linking with 0%, 5%, 10%, or 15% addition of dialdehyde starch nanocrystals (NDASs). Statistical significance is indicated with asterisks: * *p* < 0.05, ** *p* < 0.01.

The Microtox test measures the toxicity exerted on Gram-negative *Aliivibrio fischeri* bacteria. The bioluminescence of the species is directly correlated to its metabolism and cell viability. Upon contact with a toxic substance, the bioluminescence decreases in a dose-dependent manner. Additionally, the lyophilized bacteria are supplied with their cell walls broken, which results in higher susceptibility to toxicants. The prepared films were subjected to this test to evaluate their toxicity, and the results are summarized in Figure 8.

The toxicity values obtained in this study suggest that non-cross-linked CS-Gel film is the most toxic (non-biocompatible) material among the non-cross-linked films. It can be seen that the toxicity exerted by non-cross-linked chitosan is the lowest as compared to non-cross-linked gelatin and chitosan–gelatin films. However, such a high cell viability decrease for CS (61%) has to be connected to its antimicrobial properties exerted on *A. fischeri* used in the test, as it has been reported as non-toxic [87,88]. In the case of CS and CS-Gel films cross-linked with NDASs, the toxicity of the films decreases as compared to neat biopolymers. However, in the case of gelatin films, the addition of oxidized starch nanocrystals as the cross-linking agent induces dose-dependent toxicity, which increases with an increase in NDASs, reaching almost no viable cells at 15% NDAS addition. This stays in contrast with the toxicity study by Karami Juyani et al., who observed no apparent toxicity of gelatin films towards bone marrow stromal cells while CS films exerted such effects [89]. In the case of the CS-Gel combination, the increasing addition of the cross-linking NDASs also increases the toxicity, but even at 15% NDASs, the cell viability decrease was significantly lower than that for non-cross-linked films.

Other research groups also investigated the toxicity of other types of obtained materials using the Microtox test. Szurkowska et al. conducted preliminary biological tests on the bacteria *Allivibrio fischeri* for hydroxyapatite material co-substituted with manganese(II) and silicate ions [90]. Tammaro et al. [91] examined the potential environmental hazards of photovoltaic panels. In the work of Isidori et al. [92], in situ monitoring of gaseous pollutants at 17 sampling points in two seasons (winter and summer) was performed. However, no research group has used Microtox to analyze the toxicity of biopolymers cross-linked with dialdehyde starch nanocrystals, which is a research novelty.

#### 2.5.9. Protein Adsorption Study

**Figure 9:**
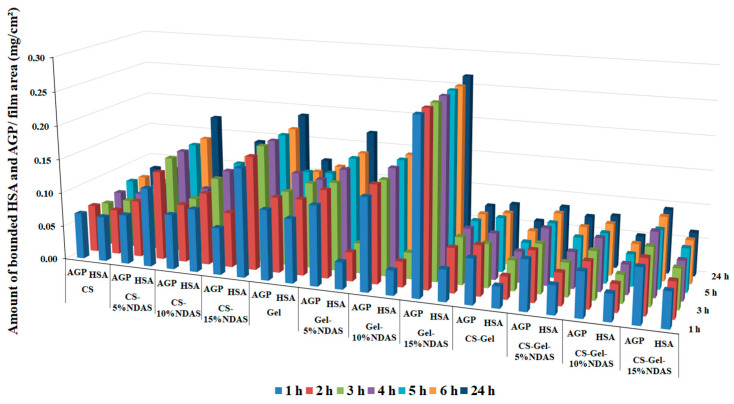
Amount of bound serum proteins AGP and HSA at the surface of obtained biopolymer films.

The study of the interaction of the obtained biopolymer films with human serum proteins allows for assessing the suitability of these materials as potential dressings. It is believed that material may be suitable for dressing purposes if it exhibits a high ability to interact with blood serum proteins [93]. When the tested films directly contact the blood, the protein is adsorbed onto the film’s surface, resulting in platelet adhesion and activation [93]. Cell adhesion requires the cell adhesion receptors to form cell-anchoring points, and protein adsorption is a crucial step during this process [94]. Human serum albumin (HSA) is the most abundant protein in the blood. It is a negative acute-phase protein. Another important serum protein is an acid glycoprotein (α-AGP), a positive acute-phase protein. The assessment of the material’s ability to interact with these proteins allows predicting whether the obtained material is a potential biomaterial for the synthesis of dressings. Therefore, in the present study, as a model, HSA and α-AGP were used to evaluate the protein adsorption behavior of the biopolymer films after different contact times (1–24 h). The obtained results are presented in Table 3 (incubation after 1, 4, and 24 h). Data in the full range of incubation timescales are presented in Supplementary Materials in Table S2 and in the diagram in Figure 9. As can be seen, all the materials show the ability to interact with blood serum proteins. The amount of deposited protein ranges from 0.031 after 1 h to 0.189 mg/cm^2^ after 24 h of exposure for HSA and from 0.065 after 1 h to 0.266 mg/cm^2^ after 24 h of exposure to α-AGP. The material that shows the ability to bind the largest amount of both proteins is non-cross-linked gelatin (Gel). The amounts of adsorbed HSA and AGP are comparable and amount to 0.111 mg/cm^2^ and 0.121 mg/cm^2^, respectively, after 24 h of exposure. The properties of gelatin significantly change after it has been cross-linked. The addition of a cross-linking agent in the form of oxidized starch nanocrystals (NDASs) causes the material to preferentially interact with acid glycoprotein from the first hour of incubation. Moreover, increasing the addition of cross-linking agent and thus the degree of cross-linking of the gelatin increases the ability of the material to bind the glycoprotein. Gelatin cross-linked with 15% NDAS addition binds almost 4 times more glycoprotein than albumin.

In the case of the non-cross-linked chitosan film, the preference for binding one of the proteins is no longer apparent. Both HSA and AGP proteins are bound in similar amounts, and the addition of NDASs as a cross-linking agent increases the amount of adsorbed HSA.

A similar situation occurs in the case of the obtained material from mixtures of both chitosan and gelatin biopolymers. For non-cross-linked films, the amounts of bound protein are very similar, with a slight predominance of the amount of glycoprotein. Taking into account that the pure chitosan film does not show such differences, it can be assumed that the greater amount of bound AGP compared to HSA is related to the addition of gelatin, which interacts preferentially with glycoprotein. The addition of NDASs as a cross-linking agent slightly increases the protein binding capacity of this material while reducing the difference between the amounts of adsorbed AGP and HSA.

The obtained results of the ability to bind HSA by the synthesized materials as potential materials for dressings do not differ from the results published in the literature. Singh and Dhiman used bovine serum albumin (BSA) to evaluate the protein adsorption on their polymer films: gum acacia-*cl*-(poly(HEMA-co-carbopol)) (GAHCP) and [poly(HEMA)-co-carbopol] (HCP), where HEMA is 2-hydroxyethyl methacrylate. Researchers observed that after 24 h, GAHCP hydrogel films and HCP films showed albumin adsorption of 0.19 ± 0.02 mg/cm^2^ and 0.24 ± 0.02 mg/cm^2^, respectively [95]. It should be emphasized that no data on the study of AGP binding capacity of biomaterials or data for similar systems tested with HSA were found in the literature.

## 3. Materials and Methods

### 3.1. Materials

Corn starch, chitosan (low molecular weight: MW = 50 kDa, deacetylation degree = 75–85%), sodium periodate, diiodomethane (pure for analysis), glycerol, human serum albumin, and acid glycoprotein were purchased from Sigma-Aldrich (St. Louis, MO, USA) and used without further purification. Acetic acid, sodium hydroxide, concentrated hydrochloric acid (35%), concentrated sulfuric acid (96%), acetone, and phosphate-buffered saline (PBS, pH = 7.4) were purchased from Avantor Performance Materials (Gliwice, Poland). Gelatin was purchased from CHEMPUR (Piekary Slaskie, Poland)—20 mesh pure. Diluent solution 2% NaCl and bacteria *A. fischeri* for toxicity assessment were supplied by the producer of Microtox (Modern Water, Cambridge, UK).

### 3.2. Starch Nanocrystal Synthesis

The starch nanocrystals were prepared by the hydrolysis process reported previously [96]. Corn starch was hydrolyzed under constant magnetic stirring for five days at 40 °C in a 3.16 M aqueous H_2_SO_4_ solution. After the hydrolysis, the nanocrystals were separated from the acid by centrifugation for 10 minutes at 8000 rpm at 4 °C. Subsequent washing and centrifugation with distilled water were performed until neutrality of the eluent and pH were controlled by litmus paper. A homogeneous dispersion of starch nanocrystals was obtained using an Ultra Turrax T25 homogenizer for 5 min at 13,500 rpm. The starch nanocrystals were dried at room temperature for 48 h.

### 3.3. Dialdehyde Starch Nanocrystal Synthesis

Previously obtained starch nanocrystals (1.5 g) were dissolved in deionized water (30 mL). Subsequently, sodium periodate (0.7 M) with three different weight ratios of oxidant to starch nanocrystals (0.5:1, 0.7:1, and 1:1) was added to the starch nanocrystal suspension. The flask was covered with aluminum foil to prevent light-induced decomposition of sodium periodate. The reaction mixture was stirred with a magnetic stirrer (IKA, Staufen, Germany) in an oil bath at 40 °C for 3 h. After the reaction was complete, the appropriate quantity of acetone was added, and white amorphous powder was formed immediately. Oxidized starch nanocrystals were isolated by filtration, washed with deionized water, and dried at room temperature for 24 h.

### 3.4. Biopolymer Preparation

Chitosan and gelatin were separately dissolved in acetic acid (C = 1% m/m) to obtain 1% solutions. Both biopolymers were mixed in a volume ratio of 1:1. Chitosan, gelatin, and their blend were mixed with the desired amount of oxidized starch nanocrystals (5%, 10%, and 15%). This amount is relative to dry protein/polysaccharide weight. The mixing of blends was carried out for 2 h with magnetic stirring. Next, all solutions were poured onto the leveled glass plates to allow the solvent to evaporate. Evaporation time was five days at room temperature for the volume 10 mL solutions of polysaccharide and mixture.

### 3.5. Characterization of Starch Nanocrystals, Dialdehyde Starch Nanocrystals, and Cross-Linked Biopolymers

#### 3.5.1. Determination of the Content of Aldehyde Groups

The aldehyde group content of the samples was determined by acid–base titration [56]. Oxidized starch nanocrystals (0.10 g) and 5 mL 0.25 M sodium hydroxide were added to the Erlenmeyer flask. The mixture was heated in a water bath at 70 °C until the sample was dissolved, and then it was cooled with cold water. Standardized 0.25 M hydrochloric acid (7.5 mL) and distilled water (15 mL) were added. Then, phenolphthalein was added, and the solution was titrated by 0.25 M sodium hydroxide. The procedure was repeated three times. The percentage of dialdehyde units is given by Equation (1):

(1) $$Aldehyde\;content,\;\%=\frac{C_{1}V_{1}\;-\;C_{2}V_{2}}{W}\times 161\times 100\%$$

where *C*_1_ and *C*_2_ (mol/L) represent the concentrations of NaOH and HCl solutions, respectively; *V*_1_ and *V*_2_ (dm^3^) represent the volume of NaOH and HCl solutions, respectively; *W* is the weight of the sample (g); and 161 (g/mol) is the molecular weight of the repeating unit in dialdehyde starch nanocrystals.

#### 3.5.2. Particle Size Distribution Analysis

The particle size distribution was analyzed using the Malvern Panalytical NanoSight LM10 instrument (sCMOS camera, 405 nm laser) using NTA 3.2 Dev Build 3.2.16 software. Before the measurements, the material dispersions were diluted with deionized water to achieve an operating range of nanoparticle concentration. The temperature of the sample chamber was set and maintained at 25.0 ± 0.1 °C; the syringe pump infusion rate was set to 200. For each sample, three 60-second movies were recorded.

#### 3.5.3. Determination of the Cross-Linking Degree of the Films

The cross-linking degree of films was determined by the extraction method, where the insoluble matrix (“gel”) reflected the cross-linked fraction of films, and the non-cross-linked fraction was fully dissolved in the solvent [97]. The acetic acid solution (5%) was chosen as a suitable solvent for chitosan–gelatin-based films. The precise initial weight of each sample (*m*_1_) was determined before measurement. Then, all samples were put in an acetic acid solution and were extracted at 70 °C for 24 h in an oil bath. After this treatment, the insoluble residue was dried at 60 °C for 24 h and then weighed again (*m*_2_). The degree of cross-linking was calculated by using Formula (2):

(2) $$Degree\;of\;the\;cross-linking,\;\%=\frac{m_{2}}{m_{1}}\times 100\%$$

#### 3.5.4. Contact Angle Measurement

The contact angle of polar glycerol and non-polar diiodomethane at constant temperature (27 °C) was measured on the biopolymers’ surface and cross-linked biofilms. The measure was performed with a DSA10 goniometer (Kruss GmbH, Hamburg, Germany) equipped with a camera. The surface free energy (γ_s_) and its polar (γ_s_^p^) and dispersive (γ_s_^d^) components were calculated by the Owens–Wendt method using the mean of at least five measurements of the contact angle of two measuring liquids [98].

#### 3.5.5. Attenuated Total Reflectance Fourier Transform Infrared (ATR-FTIR) Spectroscopy and X-ray Diffraction (XRD)

Structures of obtained materials were evaluated by attenuated total reflectance Fourier transform infrared (ATR-FTIR) spectroscopy using a Spectrum Two spectrophotometer (Perkin Elmer, Waltham, MA, USA) equipped with an ATR device (diamond crystal). The spectra were collected in the region from 4000 cm^−1^ to 400 cm^−1^ at a resolution of 4 cm^−1^ and with 64 scans at room temperature. 

X-ray diffraction (XRD) measurements were carried out in an X’PertPRO diffractometer (Malvern Panalytical, Almelo, Holland) with CuKα radiation (λ = 1.540 Å). All samples were recorded through the 2θ range of 5–120° with a step size of 0.008° at room temperature.

#### 3.5.6. Thermal Analysis

Thermal gravimetric analysis of dialdehyde starch nanocrystals, starch nanocrystals, and cross-linked biofilms was accomplished on a TA Instruments (SDT 2960 Simultaneous DSC-TGA, New Castle, DE, USA) thermogravimetric analyzer. TGA measurements were performed at a 10 °C/min heating rate in the atmosphere of nitrogen in the range from ambient to 600 °C.

#### 3.5.7. Scanning Electron Microscopy (SEM) and Atomic Force Microscopy (AFM)

Scanning electron microscopy (SEM) analyses of oxidized starch nanocrystals, starch nanocrystals, and cross-linked biofilms were carried out using a 1430 VP LEO (Electron Microscopy Ltd., Cambridge, UK) at an acceleration voltage of 20 kV. The samples were covered with a thin layer of gold before observation, and images were captured at different magnifications.

Topographic images of the obtained biofilms were recorded by the atomic force microscopy (MultiMode Nanoscope IIIa Veeco Metrology Inc., Santa Barbara, CA, USA) technique. Surface images were acquired using a scan width of 1 μm with a scan rate of 1.97 Hz. The samples were scanned with 5 μm × 5 μm areas and analyzed using NanoScope Analysis software. Roughness parameters, namely arithmetic mean, R_a_; root mean square, R_q_; and the highest peak value, R_max_, were determined.

#### 3.5.8. Mechanical Properties

In order to perform mechanical testing, the samples were cut with initial dimensions of 50 mm in length and 4.5 mm in width. The samples were inserted between the machine limbs and stretched to break using an EZ-Test E2-LX Shimadzu texture analyzer (Shimadzu, Kyoto, Japan). The measurements were carried out at the speed of 20 mm/min using a 50 N load head. The tensile strength, Young’s modulus, and elongation at break for average values of 5 measurements were obtained.

#### 3.5.9. Swelling Ability

The conventional gravimetric methods were used for the determination of the swelling ability [99]. The weight samples were immersed in phosphate-buffered saline (PBS) solution (pH = 7.4) and removed at regular time intervals. The excess buffer on the surface was wiped off, and the samples were weighed until equilibrium was reached. The swelling ability was determined as follows (3):

(3) $$Swelling\;ability,\;\%=\frac{m_{s}\;-\;m_{d}}{m_{d}}\times 100\%$$

where *m_s_* and *m_d_* denote the weights of swollen and dry samples, respectively.

#### 3.5.10. Toxicity Assessment

The materials prepared in the study were then assessed in terms of the acute toxicity in the Microtox test. Slight modifications were applied to the standard 81.9% screening test procedure for the testing of films [56]. Briefly, after registering the bioluminescence of the *A. fischeri* bacteria directly before the addition of the sample (time t = 0), 900 µL of 2% sodium chloride solution (Microtox Diluent; Modern Water, Cambridge, UK) precooled to 15 °C was added, and into such a resulting bacterial suspension, a film fragment was submerged. Next, the bioluminescence emitted by the bacteria at 490 nm was registered using Microtox M500 5 minutes and 15 minutes after the start of the exposure to the tested films [100]. The supplied software, Modern Water MicrotoxOmni 4.2 (Modern Water, Cambridge, UK), was used to calculate the percentage effect (toxicity) exerted by the materials. All the materials were tested in triplicate.

#### 3.5.11. Protein Adsorption Study

Fluorescence emission spectra were obtained on a Jasco FP-8300 spectrofluorometer (Jasco, Tokyo, Japan). Fluorescence spectra were recorded at 298 K, ranging from 290 to 400 nm at excitation wavelength 280 nm for human serum albumin (HSA) (Figure S4) and from 300 nm to 400 nm at excitation wavelength 289 nm for α1-acid glycoprotein (AGP) (Figure S5). Spectrum registration range was 285–400 nm for HSA and 300–400 nm for AGP, scanning speed was 100 nm/min, and Em/Ex bandwidth was 2.5 nm/5 nm. A stock solution of human serum albumin and α1-acid glycoprotein in phosphate buffer (PBS, pH = 7.4, 50 mM) at a concentration of 10 µM was used to prepare a protein standard curve. From the stock protein solution, solutions in PBS, pH = 7.4, 50 mM were prepared with the following concentrations: 1 μM, 2 μM, 3 μM, 4 μM, 5 μM, 6 μM, 7 μM, 8 μM, and 9 μM. Spectra were recorded for each protein solution by excitation at 280 nm for HSA and 289 nm for AGP, and a standard curve was plotted (R^2^ = 0.99).

An albumin solution and α1-acid glycoprotein were prepared in phosphate buffer pH = 7.4 (50 mM) at concentrations of 6.62 μM and 9.98 μM, respectively. Further, cut biofilms with a size of 2 × 2 cm were placed in Eppendorf tubes (Eppendorf, Hamburg, Germany). Two milliliters of the protein suspension were then added to each sample of the biofilms and incubated at 36 °C at 600 rpm. For the supernatant from each tube, fluorescence spectra were recorded by excitation at 280 nm at intervals of 1 h, 2 h, 3 h, 4 h, 5 h, 6 h, and 24 h from the start of incubation. The measurement was repeated three times.

## 4. Conclusions

In summary, novel biopolymer films based on fully natural and biocompatible materials, gelatin, chitosan, gelatin–chitosan, and NDASs, were prepared by solvent evaporation. The cross-linking agent was obtained by periodate oxidization of starch nanocrystals and characterized by ATR-FTIR, SEM images, NTA, and XRD analysis. Schiff’s base bond between NDASs and biopolymers was created. The highest degree of cross-linking was observed for chitosan–gelatin with a 15% addition of NDASs. All the prepared cross-linked films are homogeneous. The values of surface free energy polar component and contact angle for prepared materials allow stating that materials cross-linked with dialdehyde starch nanocrystals are promising for biomedical applications due to the nature of the surface. Creating a new cross-linking network between polysaccharides and oxidized starch nanocrystals leads to better mechanical properties. The swelling ability of all cross-linked biopolymer materials increased with an increase in the cross-linking agent content in the polysaccharide composition. The addition of chitosan into gelatin films caused the lowest toxicity effect on the surface compared to pristine gelatin films. All obtained materials can interact with blood serum proteins: HSA and AGP. Cross-linking of materials with NDASs increases the ability of these interactions, which is beneficial for biomedical applications of materials. Moreover, it has been noticed that NDAS cross-linked gelatin films preferentially bind glycoprotein, which can be used, for example, in pharmaceutical analysis, apart from their use in dressing materials. It can be assumed that the films’ properties were improved compared to the properties exhibited by samples without NDASs. The proposed materials can be applied as thin biofilms in biomedical applications.

## Figures and Tables

**Table 1:** Thermal parameters of neat chitosan (CS); gelatin (Gel); chitosan–gelatin (CS-Gel, 1:1); and these films cross-linked by 5%, 10%, and 15% addition of NDASs (TGA-DTG analysis in a nitrogen atmosphere).

Sample	First Stage		Second Stage			Third Stage			Residue 600 °C (%)
	T_max_ (°C)	Δm (%)	T_o_ (°C)	T_max_ (°C)	Δm (%)	T_o_ (°C)	T_max_ (°C)	Δm (%)	
CS	30, 60	8	-	-	-	145	286	51	40
CS-5%NDAS	22, 53	13	134	145	3	162	274	49	34
CS-10%NDAS	31, 52	13	121	142	4	165	277	50	33
CS-15%NDAS	32, 55	12	125	141	4	161	258	47	37
Gel	34, 56	9	-	-	-	168	307	62	27
Gel-5%NDAS	38, 61	11	141	163	3	175	312	64	23
Gel-10%NDAS	35, 59	8	138	164	3	197	312	58	30
Gel-15%NDAS	37, 60	9	124	147	4	185	306	55	32
CS-Gel	26, 70	15	-	-	-	152	290	57	28
CS-Gel-5%NDAS	38, 57	13	135	150	2	168	284	54	31
CS-Gel-10%NDAS	32, 57	12	134	148	3	168	285	51	34
CS-Gel-15%NDAS	33, 57	11	131	144	4	165	270	51	34

**Table 2:** The roughness parameters of chitosan, gelatin, and chitosan–gelatin films cross-linked with NDASs.

Roughness Parameters (nm)			Sample	
	CS	CS-5%NDAS	CS-10%NDAS	CS-15%NDAS
R_q_	9.24	1.73	2.29	6.20
R_a_	7.49	1.34	1.78	4.90
R_max_	89.9	28.1	31.3	33.9
	**Gel**	**Gel-5%NDAS**	**Gel-10%NDAS**	**Gel-15%NDAS**
R_q_	1.83	2.48	3.26	4.70
R_a_	1.48	1.61	2.36	2.64
R_max_	10.2	33.6	86.7	106.0
	**CS-Gel**	**CS-Gel-5%NDAS**	**CS-Gel-10%NDAS**	**CS-Gel-15%NDAS**
R_q_	4.65	3.16	2.06	1.45
R_a_	3.14	2.54	1.58	1.07
R_max_	65.6	27.1	18.3	14.8

**Table 3:** Amount of protein bound on the surface of biopolymers in mg of protein per 1 cm^2^ of biopolymer film, after 1, 4, and 24 h of incubation.

Amount of Adsorbed Serum Protein (mg/cm^2^)	Incubation Time (h)	Sample			
		CS	CS-5%NDAS	CS-10%NDAS	CS-15%NDAS
HSA	1	0.067	0.116	0.093	0.159
	4	0.067	0.131	0.106	0.167
	24	0.072	0.144	0.119	0.174
AGP	1	0.068	0.073	0.081	0.069
	4	0.070	0.084	0.086	0.081
	24	0.064	0.082	0.085	0.081
		**Gel**	**Gel-5%NDAS**	**Gel-10%NDAS**	**Gel-15%NDAS**
HSA	1	0.094	0.039	0.036	0.046
	4	0.112	0.042	0.037	0.065
	24	0.126	0.044	0.039	0.069
AGP	1	0.103	0.117	0.136	0.255
	4	0.111	0.129	0.144	0.256
	24	0.111	0.130	0.141	0.256
		**CS-Gel**	**CS-Gel-5%NDAS**	**CS-Gel-10%NDAS**	**CS-Gel-15%NDAS**
HSA	1	0.031	0.042	0.039	0.052
	4	0.033	0.047	0.040	0.053
	24	0.044	0.049	0.041	0.058
AGP	1	0.066	0.073	0.065	0.079
	4	0.073	0.074	0.067	0.080
	24	0.064	0.072	0.070	0.084

## Data Availability

All data generated or analyzed during this study are included in this manuscript and its Supplementary Materials.

## Supplementary Materials

The following supporting information can be downloaded at: https://www.mdpi.com/article/10.3390/ijms23147652/s1.
