# Supplementary material for: Dialdehyde Starch Nanocrystals as a Novel Cross-Linker for Biomaterials Able to Interact with Human Serum Proteins
Source: Int J Mol Sci. 2022 Jul 11;23(14):7652. doi: 10.3390/ijms23147652 (PMC9320567; doi:10.3390/ijms23147652)
Supplement: Supplementary file 1 [file ijms-23-07652-s001.zip › ijms-1804981-supplementary.pdf]

Supplementary Materials

Dialdehyde starch nanocrystals as a novel cross-linker for biomaterials able to interaction with human serum proteins

Katarzyna Wegrzynowska-Drzymalska [1], Kinga Mylkie [1], Pawel Nowak [1], Dariusz T. Mlynarczyk [2], Dorota Chelminiak-Dudkiewicz [1], Halina Kaczmarek [1], Tomasz Goslinski [2], and Marta Ziegler-Borowska [1,*]

[1] Department of Biomedical Chemistry and Polymer Science, Faculty of Chemistry, Nicolaus Copernicus University in Torun, Gagarina 7, 87-100 Torun, Poland; kasiawd@doktorant.umk.pl (K.W.-D.); kinga.mylkie@doktorant.umk.pl (K.M.); nowak19981411@wp.pl (P.N); dorotachd@umk.pl (D.C.-D.); halina@umk.pl (H.K.)

[2] Chair and Department of Chemical Technology of Drugs, Poznan University of Medical Sciences, Grunwaldzka 6, 60-780 Poznan, Poland; mlynarczykd@ump.edu.pl (D.T.M.), tomasz.goslinski@ump.edu.pl (T.G.)

* Correspondence: martaz@umk.pl (M.Z.-B.)

Figure S1. Images of all obtained samples. 3

Table S1. The average contact angle for glycerin and diiodomethane for films of chitosan (CS), gelatin (Gel), and chitosan-gelatin (CS-Gel) cross-linked by 5%, 10% and 15% adding of cross-linker (NDAS). 4

Figure S2. The TGA-DTG curves of (a) chitosan, (b) gelatin, and (c) chitosan-gelatin (1:1) cross-linked with 5%, 10%, and 15% of NDAS.. 5

Figure S3. AFM images in 2D scale of (a) chitosan films cross-linked by (d) 5% NDAS, (g) 10% NDAS, and (j) 15% NDAS, (b) gelatin film cross-linked by (e) 5% NDAS, (h) 10% NDAS, and (k) 15% NDAS, and (c) chitosan-gelatin films cross-linked by (f) 5% NDAS, (i) 10% NDAS, and (l) 15% NDAS. 6

Table S2. Amount of protein bound on the surface of biopolymers in mg of protein per 1 cm^2 of biopolymer film in the full range of incubation timescales.. 7

Figure S4. Emission fluorescence spectra of human serum albumin with increasing concentrations. 8

Figure S5. Emission fluorescence spectra of α1-acid glycoprotein with increasing concentrations.9

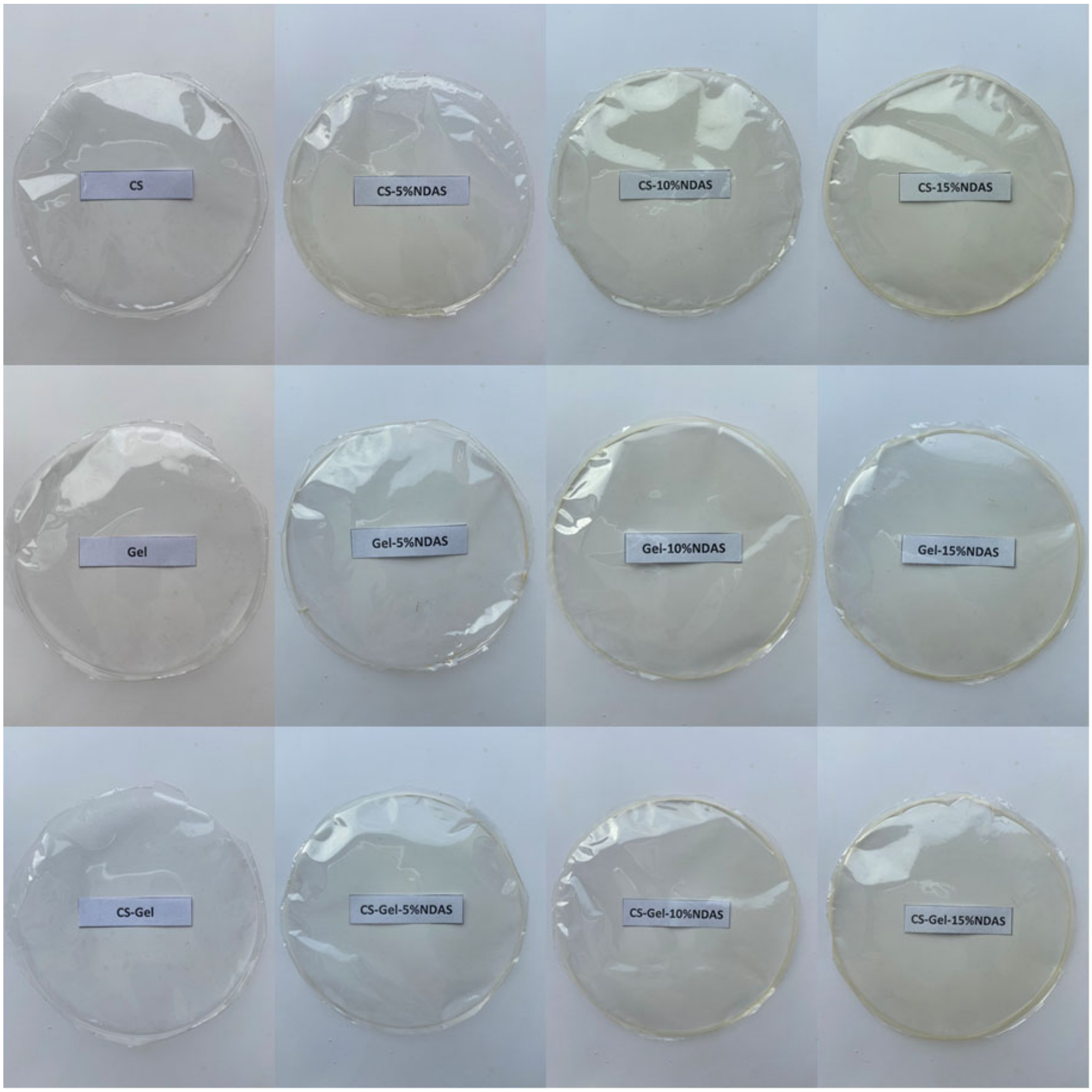

Figure S1. Images of all obtained samples.

Table S1. The average contact angle for glycerin and diiodomethane for films of chitosan (CS), gelatin (Gel), and chitosan-gelatin (CS-Gel) cross-linked by 5%, 10% and 15% adding of cross-linker (NDAS).

**Sample**	**Average Contact Angle [θ, °]**	
	**Measuring Liquid**	
	Glycerin	Diiodomethane
CS	82.0	56.0
CS-5%NDAS	79.4	55.7
CS-10%NDAS	76.6	54.6
CS-15%NDAS	76.1	49.0
Gel	76.9	44.8
Gel-5%NDAS	72.7	46.9
Gel-10%NDAS	69.4	49.0
CS-15%DAS	72.5	45.3
CS-Gel	71.6	46.8
CS-Gel-5%NDAS	75.2	49.7
CS-Gel-10%NDAS	72.7	48.0
CS-15%Glu	74.4	46.2

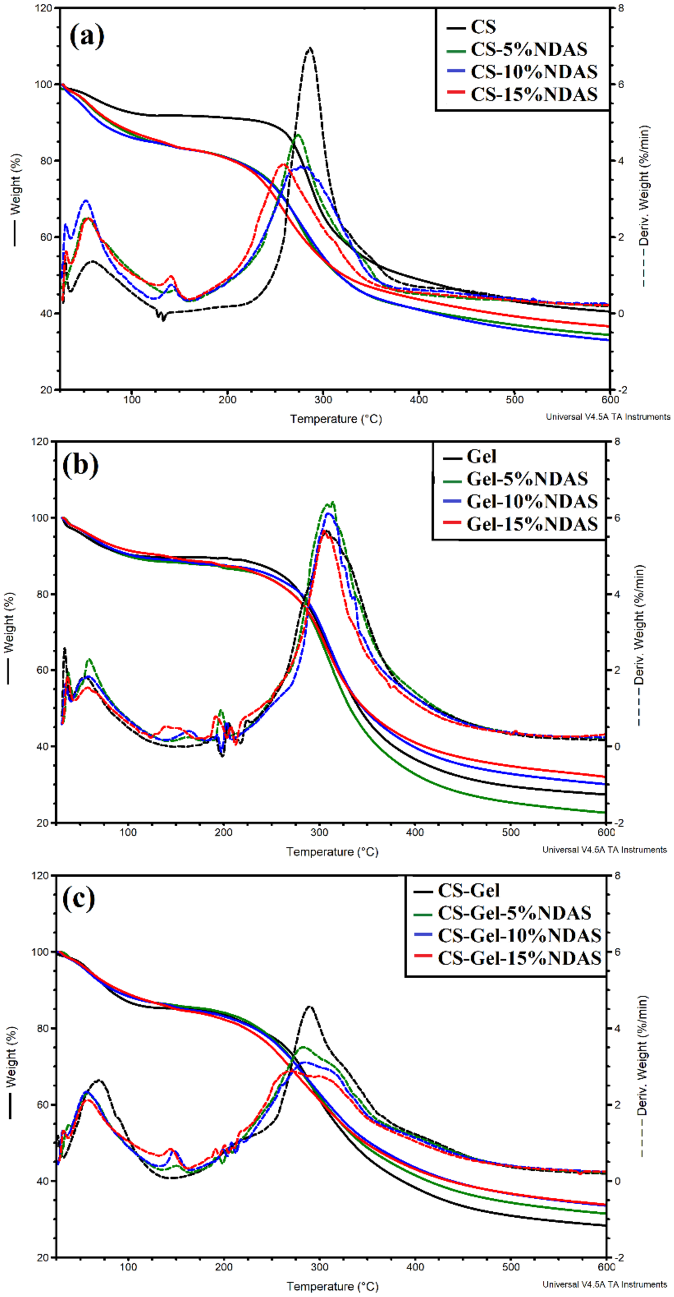

Figure S2. The TGA-DTG curves of (a) chitosan, (b) gelatin, and (c) chitosan-gelatin (1:1) cross-linked with 5%, 10%, and 15% of NDAS.

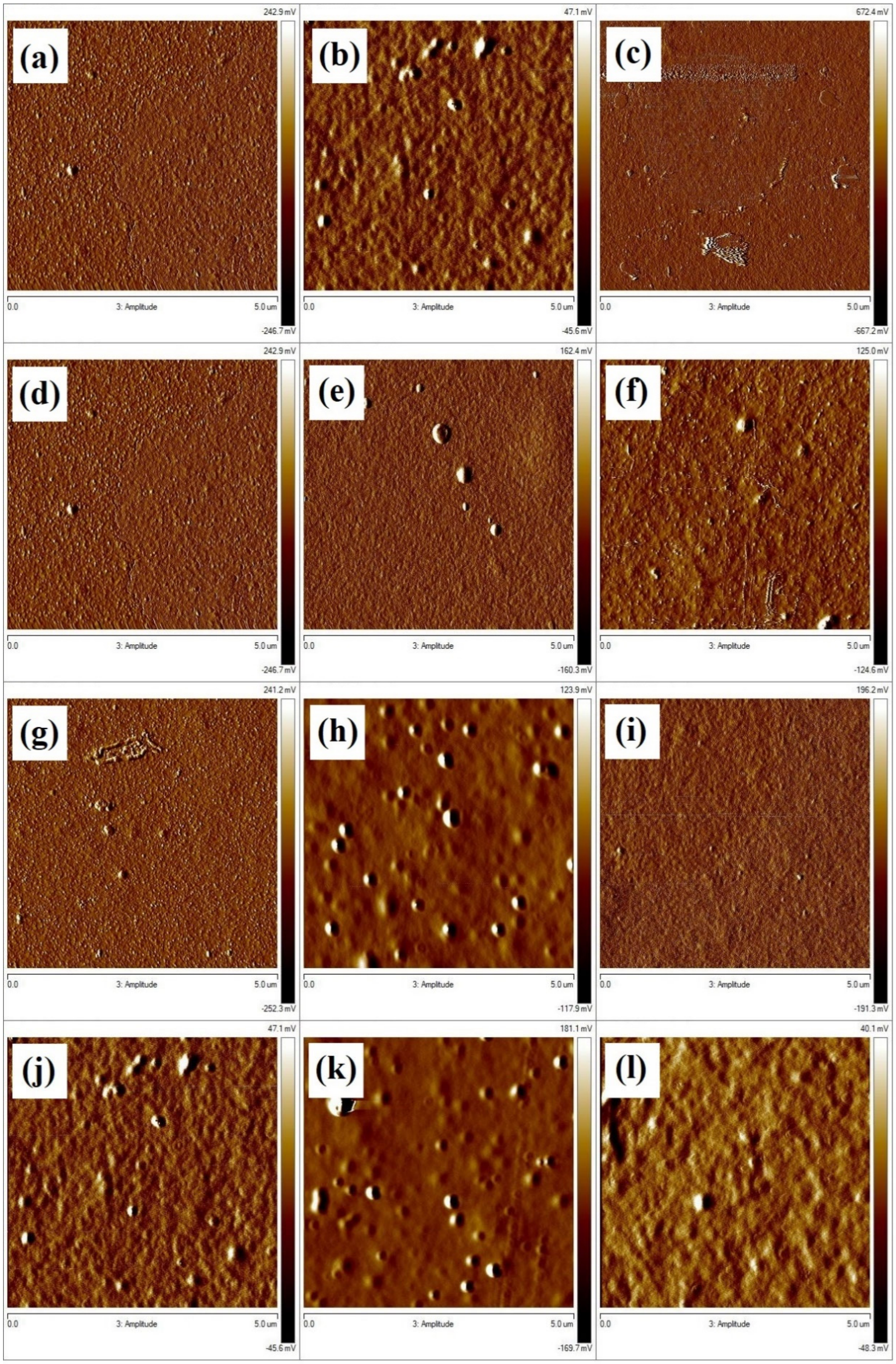

Figure S3. AFM images in 2D scale of (a) chitosan films cross-linked by (d) 5% NDAS, (g) 10% NDAS, and (j) 15% NDAS, (b) gelatin film cross-linked by (e) 5% NDAS, (h) 10% NDAS, and (k) 15% NDAS, and (c) chitosan-gelatin films cross-linked by (f) 5% NDAS, (i) 10% NDAS, and (l) 15% NDAS.

Table S2. Amount of protein bound on the surface of biopolymers in mg of protein per 1 cm^2 of biopolymer film in the full range of incubation timescales.

Sample	Incubation time [h]													
	1h		2h		3h		4h		5h		6h		24h	
	Amount of adsorbed serum protein [mg/cm^2]													
	HSA	AGP	HSA	AGP	HSA	AGP	HSA	AGP	HSA	AGP	HSA	AGP	HSA	AGP
**CS**	0.067	0.068	0.067	0.070	0.072	0.064	0.072	0.071	0.073	0.080	0.073	0.077	0.084	0.083
**CS-5%NDAS**	0.116	0.073	0.131	0.084	0.144	0.082	0.145	0.090	0.147	0.080	0.148	0.094	0.174	0.108
**CS-10%NDAS**	0.093	0.081	0.106	0.086	0.119	0.085	0.121	0.091	0.124	0.090	0.126	0.092	0.141	0.094
**CS-15%NDAS**	0.159	0.069	0.167	0.081	0.174	0.081	0.173	0.087	0.174	0.080	0.175	0.086	0.189	0.089
**Gel**	0.094	0.103	0.112	0.111	0.126	0.110	0.122	0.128	0.123	0.121	0.123	0.112	0.111	0.121
**Gel-5%NDAS**	0.039	0.117	0.042	0.129	0.044	0.130	0.045	0.140	0.045	0.148	0.047	0.147	0.051	0.170
**Gel-10%NDAS**	0.036	0.136	0.037	0.144	0.039	0.141	0.041	0.149	0.044	0.152	0.047	0.151	0.049	0.169
**Gel-15%NDAS**	0.046	0.255	0.065	0.256	0.069	0.256	0.071	0.258	0.072	0.259	0.072	0.258	0.074	0.266
**CS-Gel**	0.031	0.066	0.033	0.073	0.044	0.064	0.045	0.068	0.048	0.080	0.054	0.077	0.058	0.080
**CS-Gel-5%NDAS**	0.042	0.073	0.047	0.074	0.049	0.072	0.053	0.083	0.063	0.080	0.068	0.084	0.073	0.083
**CS-Gel-10%NDAS**	0.039	0.065	0.040	0.067	0.041	0.070	0.044	0.077	0.047	0.073	0.051	0.076	0.051	0.077
**CS-Gel-15%NDAS**	0.052	0.079	0.053	0.080	0.058	0.084	0.058	0.094	0.064	0.086	0.064	0.094	0.064	0.094

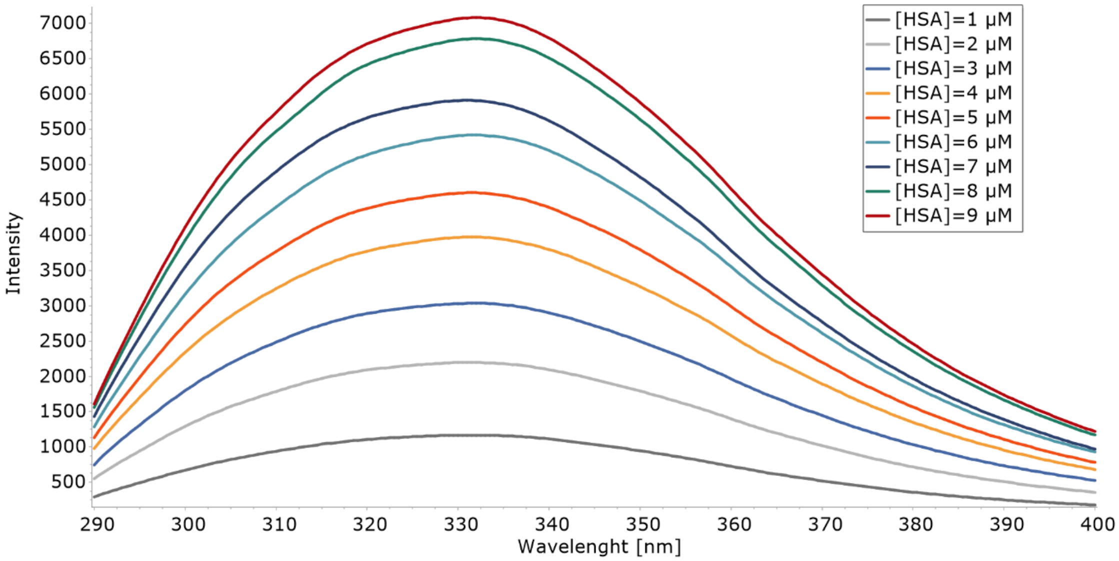

Figure S4. Emission fluorescence spectra of human serum albumin with increasing concentrations.

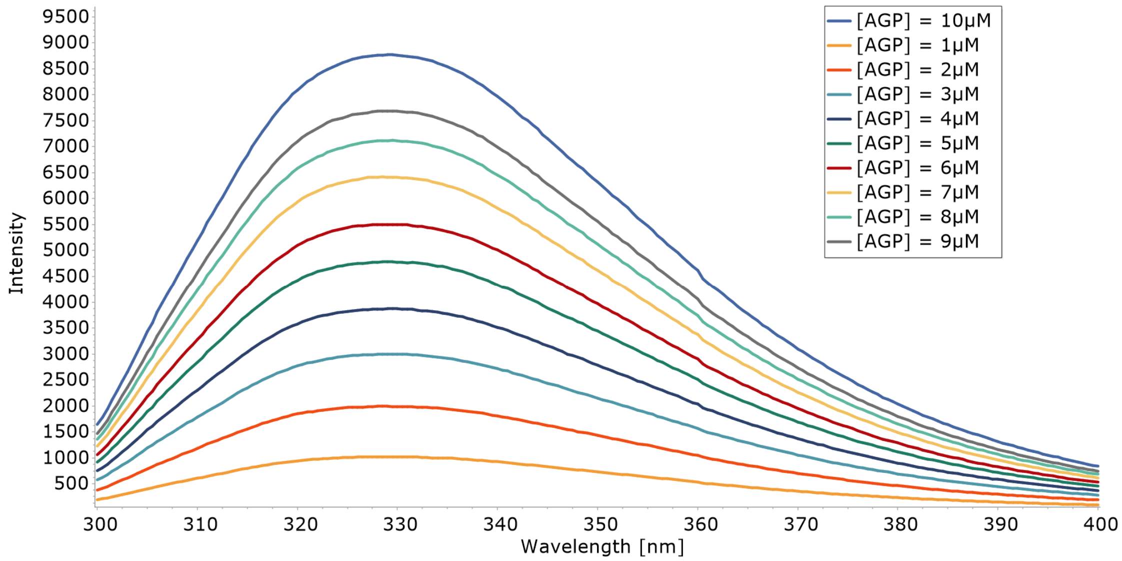

Figure S5. Emission fluorescence spectra of α1-acid glycoprotein with increasing concentrations.
